# Yellow Fever

**Published:** 1880-10

**Authors:** 


YELLOW FEVER.— This dreaded pestilence, seems to have given its usual places of rendezvous in our Southern cities, a complete go-by thus far, this season. And were it to appear now, our proximity to *frost* would *prevent* its doing a great deal of damage or harm to our people.

We have not yet so far forgotten the *teaching of nature* and *experience*, as to embrace the *hallucination*, that "frost and cold do not destroy the yellow fever germ."

This is an asseveration made in the face of ascertained *facts*, and will hardly have any other effect, than to provoke ridicule, where it may attract any notice at all.

We believe it was Dr. Franklin who said that.

Vessels large may venture more, but *little boats should keep near the shore.*

